# Salutary effects of transdermal curcumin on multiple indices of health span in rodent models of normal aging and hypertension

**DOI:** 10.1007/s11357-025-01607-8

**Published:** 2025-03-15

**Authors:** Kai Mao, Ruixuan Wang, Kateryna Karpoff, Daniel Kerr, Probal Banerjee, Joel M. Friedman, Derek M. Huffman

**Affiliations:** 1 Department of Molecular Pharmacology, Albert Einstein College of Medicine, Bronx, NY USA; 2 Department of Medicine, Albert Einstein College of Medicine, Bronx, NY USA; 3 Department of Microbiology & Immunology, Albert Einstein College of Medicine, Bronx, NY USA; 4 Institute for Aging Research, Albert Einstein College of Medicine, 1300 Morris Park Ave, Golding Building, Room 201, Bronx, NY USA; 5 The Center for Developmental Neuroscience, CUNY College of Staten Island, Staten Island, NY 10314 USA; 6 Department of Chemistry, CUNY College of Staten Island, Staten Island, NY 10314 USA; 7 Vascarta Inc, Summit, NJ 07901 USA

**Keywords:** Curcumin, Hypertension, Aging, Frailty, Inflammation

## Abstract

Geroscience has helped to usher in a new and exciting era of aging drug development and evaluation of novel and repurposed agents, as well as natural compounds purported to target one or more aging hallmarks. Among the latter, curcumin has long been pursued as a promising strategy but has failed to provide convincing evidence in human trials. Oral intake is the typical route of administration tested for the vast majority of gerotherapeutic candidates, including curcumin, but efficacy is dependent upon good oral bioavailability and pharmacokinetics. However, unlike FDA-approved oral medications, many natural compounds, such as curcumin, have poor oral bioavailability, which may explain their limited success in translation. To overcome these inherent limitations, we tested a novel solvent-based formulation of concentrated curcumin (VASCEPTOR®), developed for effective skin penetration and delivery of high amounts of bioactive curcuminoids directly to the circulation on aging and age-related conditions. We demonstrate that short-term topical treatment (7.5 mg per dose) with VASCEPTOR® twice per week can improve both vascular health in a rat model of hypertension, while a late-life intervention in aged mice improves multiple indices of health span, including improved exercise tolerance, motor coordination, diastolic function (*p* < 0.05), a reduction in frailty status (*p* < 0.05) and expression of some age-related markers in tissues, particular heart and kidney. Thus, these data suggest that the therapeutic potential of curcumin can potentially be dramatically enhanced by topical delivery and, along with other promising candidates, should be prioritized for further development, testing and deployment to potentially target some manifestations of aging in humans.

## Introduction

Recent progress in geroscience has led to new insights regarding the putative underlying drivers of the aging process, which are conceptualized as part of an evolving framework of 12 aging hallmarks [1]. These developments have in turn helped to galvanize interest in identifying aging drugs and compounds to target one or more of these hallmarks to potentially slow aging [2–6]. The National Institute on Aging (NIA) Intervention Testing Program (ITP), which has been a leader in this effort, has identified several compounds, highlighted by rapamycin [6], that can improve lifespan in heterogenous mice [7–9]. A requirement of compounds screened in this program is that the tested agent must be able to be delivered orally via a formulated diet. This in turn necessitates that compounds have good oral bioavailability, which is not always the case for many promising agents, including rapamycin, which led to microencapsulation strategies to improve its absorption [6].

One example of a potentially promising gerotherapeutic that has failed to translate, including in ITP trials [10], is curcumin. Curcuminoids are ancient, natural polyphenol phytochemicals with exceptional therapeutic promise for preventing and treating the clinical consequences of chronic and acute pro-inflammatory triggers that underlie many age-related manifestations [11–15]. These compounds can modulate a plethora of pathways to exert potent anti-inflammatory, antioxidant and pro-metabolic effects, which could be harnessed to combat a host of age-related conditions, which has motivated several trials to test its efficacy for various ailments [16, 17] and health parameters [18]. Despite extensive and promising preclinical studies with high-dose administration, translation of curcuminoids has suffered from limitations in drug (oral) delivery, which is stymied by low solubility, low uptake from the gut, modifications by gut organisms and first pass degradation by the liver to undermine efficacy [19–22]. Attempts to enhance oral bioavailability, such as by adding piperine or nanoemulsions [12], have increased systemic uptake, but have not sufficiently enhanced therapeutic efficacy.

In order to overcome these inherent limitations, we have evaluated a novel solvent-based formulation of concentrated curcuminoids (VASCEPTOR®) [23], capable of skin penetration and hence able to bypass many of the obstacles involved in oral intake to achieve delivery of high amounts of bioactive curcuminoids directly to the circulation and tissues. Moreover, we demonstrate that short-term dosing with VASCEPTOR® twice per week can improve both vascular health in a rat model of hypertension and exhibits a myriad of benefits in a mouse model of normal aging. Thus, these data suggest that the therapeutic potential of natural compounds and compatible small molecules can be greatly enhanced by transdermal delivery, opening the door for other promising gerotherapeutic agents known to suffer from poor uptake, of which there are many, for further development and testing to target the manifestations of aging.

## Methods

### Animals

For pharmacokinetic (Pk) studies in mice, young C57BL/6 J males (Jax strain 000664) were used. For aging studies, CB6F1 male mice were obtained from the NIA Aged Rodent Colony at 20 months and group housed four per cage. For Pk and blood pressure studies in rats, 4-month-old male Sprague Dawley rats were obtained from Charles River (strain 001) and housed two per cage. All animals were maintained under standard temperature (~ 22 °C) and humidity-controlled conditions and a 14L:10D photoperiod and provided ad libitum access to water and standard rodent chow (Purina 5001). All experimental procedures performed at Einstein and CUNY were approved by the respective Institutional Animal Care and Use Committees.

### PK studies and curcumin detection

To initially assess Pk properties of VASCEPTOR®, young mice were briefly anesthetized, the abdomen shaven and 200 μL curcuminoid gel topically applied. Following treatment, a subset of mice were assigned to have blood collected transcardially at either 15, 30, 60 or 180 min later (*n* = 3 per time point) into heparinized tubes, and tissues were excised for subsequent curcuminoid analysis. To compare oral versus transdermal Pk properties of VASCEPTOR®, curcuminoid formulation was administered to young rats at an equal dose either via oral gavage or transdermally applied to a shaven patch of skin on the back (~ 25 mm^2^). Following administration, a subset of rats were anesthetized and sacrificed at either 30, 60, 180 or 360 min (*n* = 3–4 per time point). Samples were then subjected to a custom extraction and HPLC protocol specifically designed for curcuminoids as described [24].

### Transdermal curcumin and health span study design

To determine the effects of a late-life transdermal curcuminoid intervention on health span, 20-month-old CB6F1 male mice were assigned to either vehicle (*n* = 18) or transdermal VASCEPTOR® (*n* = 20) treatment (7.5 mg curcuminoids per dose) on the back (shaven), twice per week for 12 weeks. To evaluate phenotypic changes, we longitudinally monitored body weight in all cohorts once per week. At ~ 22 to 23 months of age, following 2 to 3 months of treatment, a battery of health span assays were conducted, and animals were subsequently euthanized for tissue collection and subsequent analysis.

### Physical function

Gross motor coordination was assessed by the balance beam test [25]. In brief, animals were first familiarized with the testing setup by walking twice across a 4-ft plank. Animals were then challenged to transverse a 48″ long round beam of decreasing difficulty (0.5″ difficult, 0.75″ medium, 1″ easy), with light and food cues as motivation to cross, and the number of slips was counted while crossing the beam. Grip strength was also assessed by suspending mice from the tail and allowing them to clasp a 20-g weight, and the time to release (best of three trials) was recorded. Endurance capacity was determined by a single test on a treadmill (Exer 3/6, Columbus Instruments). Mice were first familiarized to the treadmill for three non-consecutive days for 5 min at a walking speed (8 m/min). Animals were then challenged with a graduated fatigue test, beginning at a 4% incline and 8 m/min for 3 min. The protocol used increased speed to 10 m/min at 3 min, 12 m/min at 4 min, 15 m/min at 5 min which was maintained until fatigue (all animals voluntarily fatigued prior to 30 min) [25, 26]. Insulin sensitivity was assessed by insulin tolerance tests (ITTs) as a proxy of metabolic health at approximately 23 months of age. In brief, mice were fasted early in the morning for approximately 1 h, and a baseline blood glucose measurement was made. Animals were then injected IP with insulin (1 mU/kg), and blood glucose levels were checked at 15, 30, 45 and 60 min after injection, as described [25–27]. Frailty index was determined at 23 months of age using a 31-point index previously described [28].

### Echocardiography

Left ventricular systolic function was assessed following ~ 10 weeks of treatment as described [25]. In brief, the Visual Sonic Vevo2100 imaging system (FUJIFILM VisualSonics Inc, Toronto, ON) was used to measure echocardiography (*n* = 8 per group), Cardiac left ventricular measurements were obtained under M-mode, and left ventricular ejection fraction (EF) and fractional shortening (FS) were correspondingly calculated.

### Cognitive and behavioral assessments

To evaluate visuospatial memory and learning, mice underwent the 1-day box maze [29, 30]. Mice were placed in a brightly lit square arena with seven false escapes and one true escape to a dark, enclosed box. Each mouse was subjected to one trial period with four subsequent testing periods. Each trial and test period lasted up to 10 min with 2-min intervals between each trial/test, in which the latency to escape and the number of false exit attempts were recorded. The elevated zero-maze assessment [30, 31] was conducted to measure anxiety-like behavior. Mice are placed in an open section of an elevated, ring-shaped, platform that is half enclosed on both sides (closed arms) and half exposed (open arms) with bumpers surrounding the entire apparatus to protect any mice falling off the platform. Once placed on an open arm, mice are allowed to roam for 5 min, in which the amount of time in the open and closed arms was recorded. More time spent in the open arms is considered to be indicative of a lower level of anxiety.

### RNA isolation and expression

Total RNA from frozen heart, kidney, cortex, liver and lung tissues was isolated using TRIzol® Reagent per the manufacturer’s instructions (Life Technologies). First-strand complementary DNA (cDNA) was synthesized with random primers using Bio-Rad iScript cDNA Synthesis Kit. All qPCR reactions were carried out using Bio-Rad Sso Advanced SYBR Green mix on a Bio-Rad CFX384 qRT-PCR Machine, and all data were normalized to cyclophilin A (*ppia*) [26, 30, 32].

### Tissue NAD+ and NADH quantification

For NAD/NADH quantification, a commercially available calorimetric assay was used (Abcam Inc; cat# ab65348). Approximately 20 mg of heart tissue was pulverized and homogenized in 200 µL of extraction buffer using a hand-held electronic homogenizer on ice. The homogenate was centrifuged at 12,000 × g for 5 min at 4 °C, and the supernatant collected. To eliminate interference from NAD-consuming enzymes, the lysate was deproteinized using a 10-kD spin column as recommended (Abcam Inc; cat#ab93349). For NADH-specific measurements, 100 µL of the sample was first heated at 60 °C for 30 min to decompose NAD+ . The plate was then incubated at room temperature for 1 h, and absorbance was measured at 450 nm. A standard curve was used to calculate NAD+ and NADH concentrations.

### Tissue 8-hydroxy-2′-deoxyguanosine 8-OHdG detection

As a proxy of DNA oxidative damage, the Cayman DNA/RNA Oxidative Damage ELISA kit (Cayman Chem; cat#589,320), which measures oxidative DNA damage marker 8-hydroxy-2′-deoxyguanosine (8-OHdG), was used. For DNA damage quantification, 50 µL of purified DNA from tissues (25 µg/µL, totaling 1250 µg) was added to a 96-well plate, and following incubations and washes were measured at 412 nm, using a standard curve to calculate the concentration of 8-hydroxy-2′-deoxyguanosine (8-OHdG) in the DNA samples.

### L-NAME challenge

To test the potential effect of VASCEPTOR® on vascular health and function, we performed a 6-week study in rats to assess the effects of treatment on blood pressure in response to an L-NAME challenge, similar as described [33, 34]. Blood pressure was measured at baseline and weekly via CODA tail cuff measures in awake animals after warming and acclimation. The study consisted of four experimental groups as follows: (1) Con Veh, (2) Con VASCEPTOR®, (3) L-NAME Veh and (4) L-NAME VASCEPTOR® (*n* = 8 per group). Initially, rats were assigned to receive either transdermally applied vehicle (Con Veh; L-NAME Veh) or 200 L curcuminoid gel (Con VASCEPTOR®; L-NAME VASCEPTOR®) prophylactically twice per week for 2 weeks. At week 3, groups 1 and 2 remained on the same regimen while groups 3 and 4 were switched to drinking water containing L-NAME for 2 weeks (40 mg/kg/day). At week 5, L-NAME groups were then switched back to normal drinking water, and blood pressure was monitored for two additional weeks to assess recovery.

### Statistics

Data were analyzed using Prism either by independent sample *t* test or two-way analysis of variance (ANOVA) for cross-sectional data, and longitudinal measures were assessed by either one or two-way ANOVA with repeated measures on time. Sidek post-hoc adjustments were performed when appropriate. Data not normally distributed were log transformed to ensure normality of distribution. All values reported are means ± standard error (SE). A *p* < 0.05 was considered to be statistically significant.

## Results

### Transdermal curcumin demonstrates superior Pk properties to oral administration

**Fig. 1:**
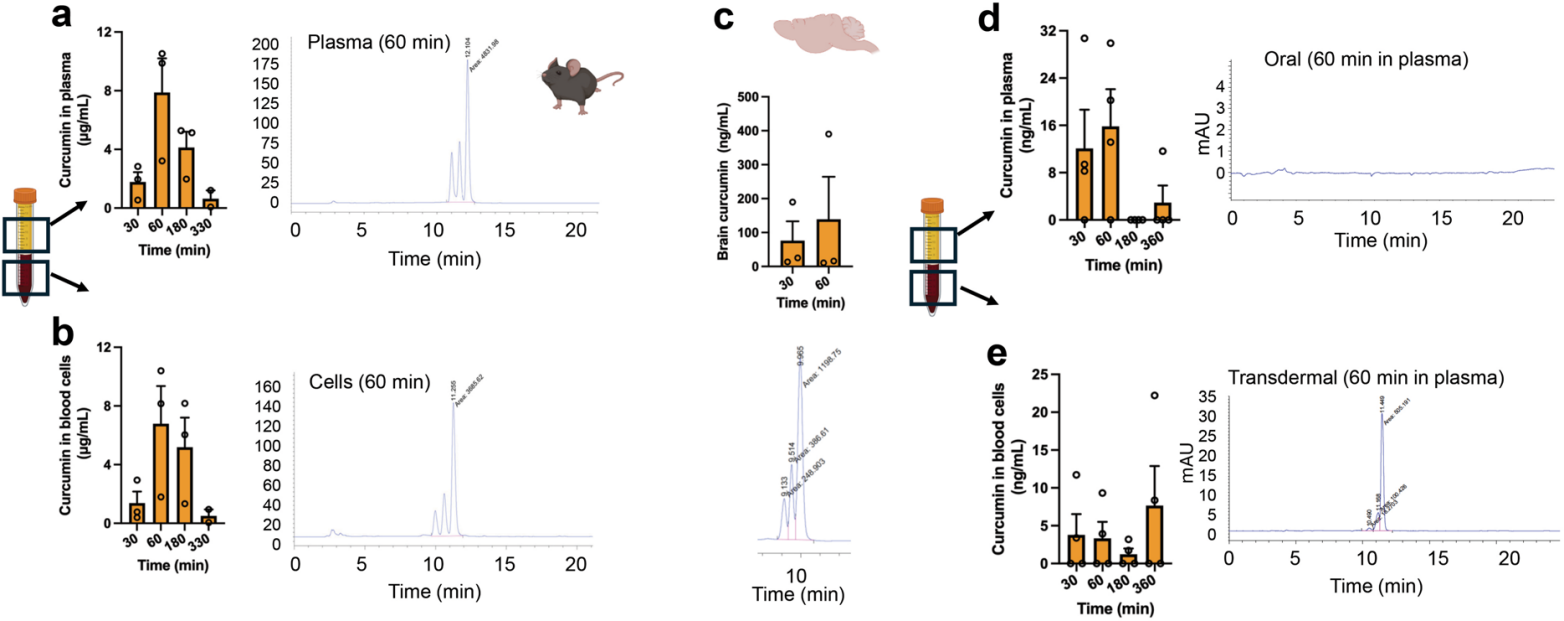
Transdermal curcumin demonstrates superior Pk properties to oral administration.** a** To initially assess Pk properties of VASCEPTOR®, 200 µL of the formulation was applied topically to the abdomen of young mice and levels assessed for up to 330 min. Plasma curcumin levels were highest (mean 7.87 µg/mL) 60 min after topical application of Vasceptor™. **b** Curcumin in the cell pellet obtained after centrifugal separation of the plasma was extracted and analyzed by HPLC. Curcumin levels in circulating cells are highest (mean 6.78 µg/mL) 60 min after topical application of VASCEPTOR™ (*n* = 3 per timepoint). **c** Curcumin levels and representative HPLC chromatogram in the brain following topical application reveal that levels can be readily detected at 60 min after administration (*n* = 3 per timepoint). **d–e** Detection of curcumin in plasma and blood cells following 200 µL (7.5 mg curcumin) VASCEPTOR® treatment via oral gavage or topically to young rats. While curcumin levels in plasma following oral gavage were undetectable, transdermal application led to curcumin levels that were readily detectible at 30 and 60 min later, and for at least 360 min after dosing in some animals (*n* = 3–4 per timepoint). Figure was generated with assistance from Biorender

To initially assess Pk properties of VASCEPTOR® applied transdermally, we applied 200 µL of curcuminoid gel topically to the abdomen of young mice and assessed curcumin levels in circulation for up to 330 min. As can be shown in Fig. 1a, curcumin was readily detectable in plasma by 30 min, peaked to ~ 300 pmol concentrations on average by 60 min and remained detectable at 330 min post application, while levels in blood cells also persisted for at least 330 min after dosing (Fig. 1b). Moreover, when brain tissue was assessed to determine the extent of curcumin penetrance into the central nervous system (CNS), curcumin levels were readily detectable at 30 (not shown) and 60 min post transdermal application (Fig. 1c). We further aimed to compare Pk of transdermal versus oral curcumin administration side-by-side via gavage or topical administration of an identical dose of VASCEPTOR® in a rat model. While levels of curcumin in circulation were undetectable following orally administered VASCEPTOR®, transdermal application led to curcumin levels that were readily detectible at 30 (not shown) and 60 min later (Fig. 1d), while levels in blood cells persisted for at least 360 min after dosing in some animals (Fig. 1e).

### Transdermal curcumin improves multiple aspects of health span

**Fig. 2:**
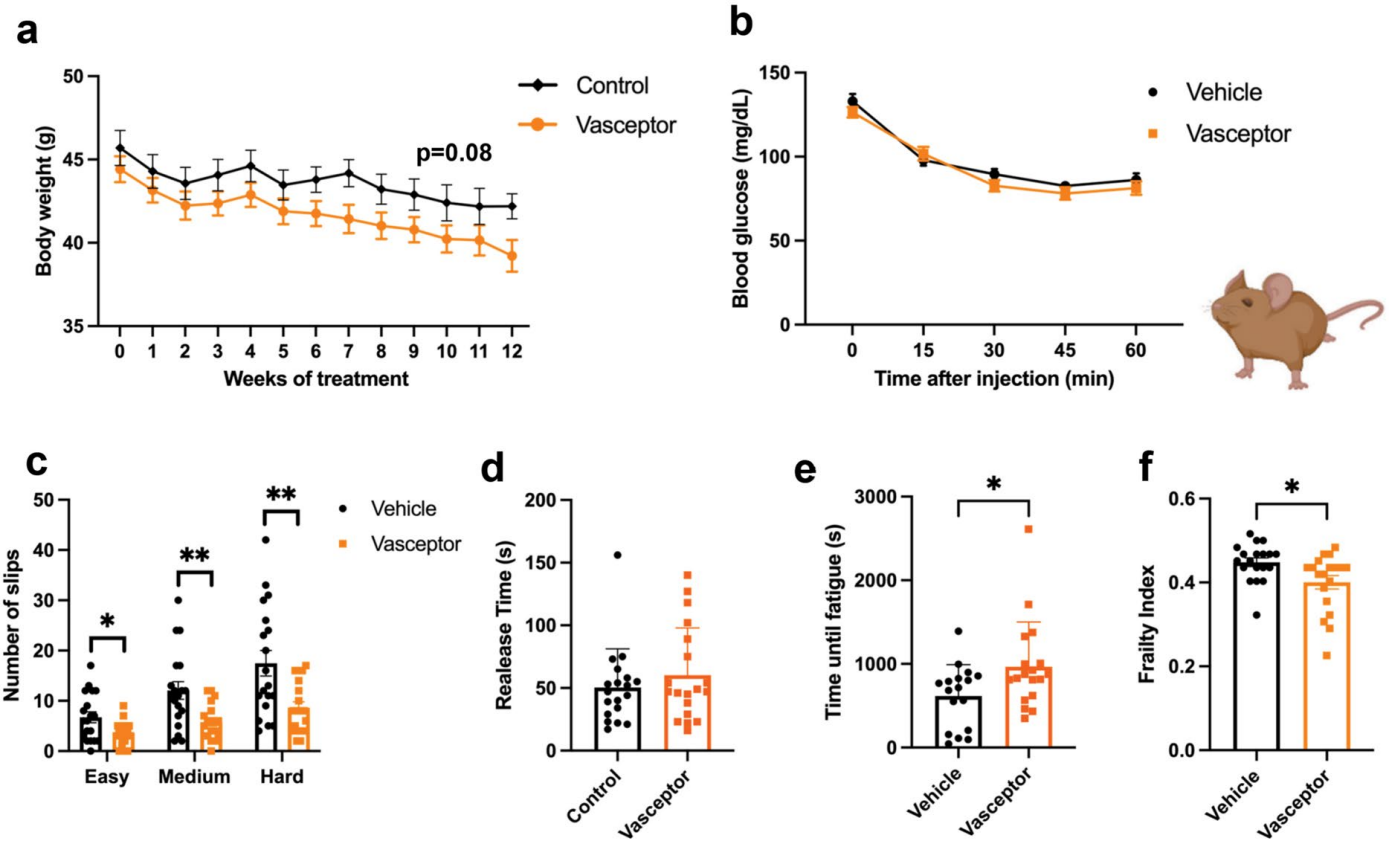
Transdermal curcumin improves multiple aspects of health span in aged mice. Aged male CB6F1 mice (20 months old) were treated topically with VASCEPTOR®, twice per week, and assessed for aspects of health span following 9–12 weeks of treatment. **a–b** As compared to vehicle-treated animals, VASCEPTOR® tended to lead to a slight, albeit non-significant reduction in body weight (Veh *n* = 18; VAS *n* = 20; *p* = 0.08), without effects on insulin sensitivity, as determined by an ITT challenge. **c–f** When assessing indicators of functional health and condition, VASCEPTOR®-treated mice had improved coordination on balance beams of increasing difficulty, including fewer slips on easy (*p* < 0.05), medium or hard difficulty (*p* < 0.01). While grip strength was not significantly different between groups, VASCEPTOR® significantly improved exercise capacity during an acute treadmill challenge (*p* = 0.0397) and led to a notably lower frailty index following 12 weeks of treatment (*p* = 0.0063). Statistics for body weight and ITT were performed via repeated measures ANOVA and other tests via independent samples *t* test. Bar and line graphs represent the mean ± S.E. *p < 0.05, **p < 0.01. Figure was generated with assistance from Biorender

To determine the gerotherapeutic potential of transdermally applied VASCEPTOR®, we performed a short-term intervention trial, treating 20-month-old male CB6F1 mice twice per week for 12 weeks. As compared to vehicle-treated animals, VASCEPTOR® led to a slight, non-significant reduction in body weight (Fig. 2a; *p* = 0.08). We further evaluated several health span parameters after 9–12 weeks of treatment. VASCEPTOR® did not alter insulin sensitivity, as determined by an ITT (Fig. 2b). However, when assessing coordination on balance beams of increasing difficulty, VASCEPTOR®-treated mice had fewer slips, and hence better performance on beams of easy (*p* < 0.05), medium or hard difficulty (Fig. 2c; *p* < 0.01). Moreover, while grip strength was not significantly different between groups (Fig. 2d), VASCEPTOR® significantly improved exercise capacity during a treadmill challenge (Fig. 2e; *p* = 0.0397). Importantly, VASCEPTOR® led to a notably lower frailty index following 12 weeks of treatment (Fig. 2f; *p* = 0.0063).

**Fig. 3:**
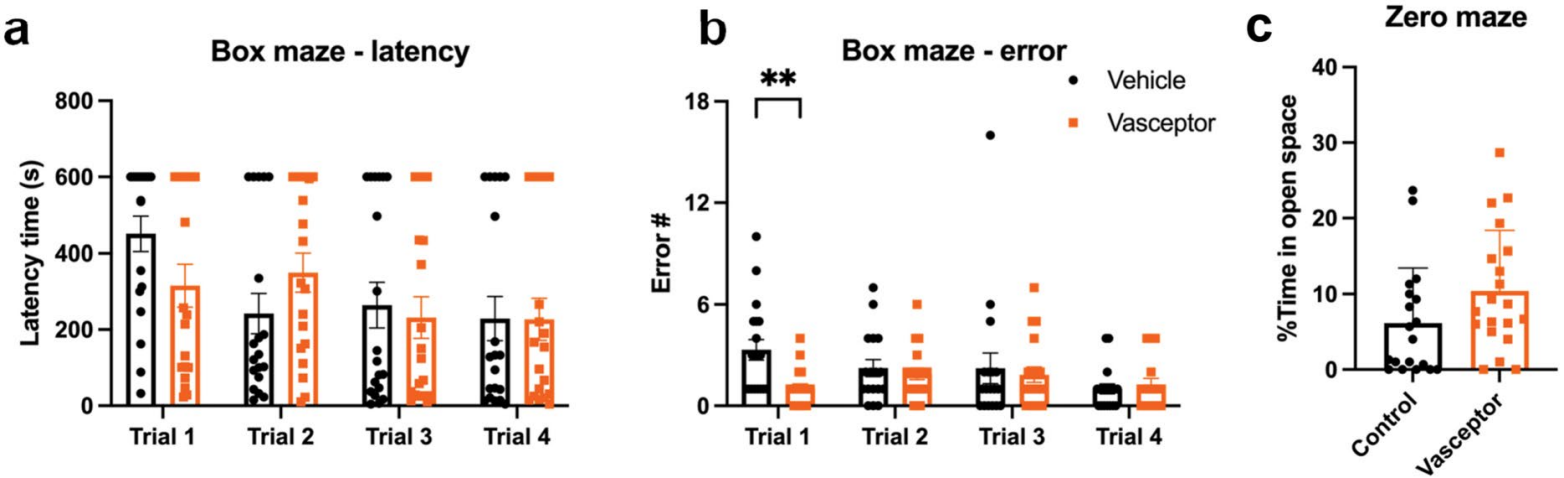
Effect of transdermal curcumin on memory and behavior in aged mice. Memory and behavior were assessed via a 1-day box maze and zero maze, respectively (Veh *n* = 18; VAS *n* = 20). **a–b** In comparing memory, a significant treat × time interaction was observed for latency (*p* = 0.041), but there were no differences between individual trials. For escape errors, a significant treat × time interaction was also observed for latency (*p* = 0.02), which was significantly reduced in VASCEPTOR®-treated mice at Trial 1, but no significant differences were observed at later trials. To assess anxiety, zero maze was performed, whereby a numerical increase in percent time spent in the open arm during the evaluation was observed in treated mice, but this was not significant (*p* = 0.09). Barnes Maze was assessed by two-way ANOVA with repeated measures on time and assessed via Sidak multiple comparisons. Zero maze was assessed via independent sample *t* tests. Bar graphs represent the mean ± S.E. **p < 0.01

We further assessed effects on memory and behavior via a 1-day box maze and zero maze, respectively. VASCEPTOR®-treated mice tended to have a lower escape latency (Fig. 3a) and fewer errors in locating the escape (Fig. 3b; *p* = 0.008) at Trial 1, but no differences were observed for learning in subsequent trials. This was accompanied by a numerical increase in percent time spent in the open arm on Zero maze (Fig. 3c; *p* = 0.09).

### Transdermal curcumin improves systolic function and reduces expression of fetal genes in myocardium

**Fig. 4:**
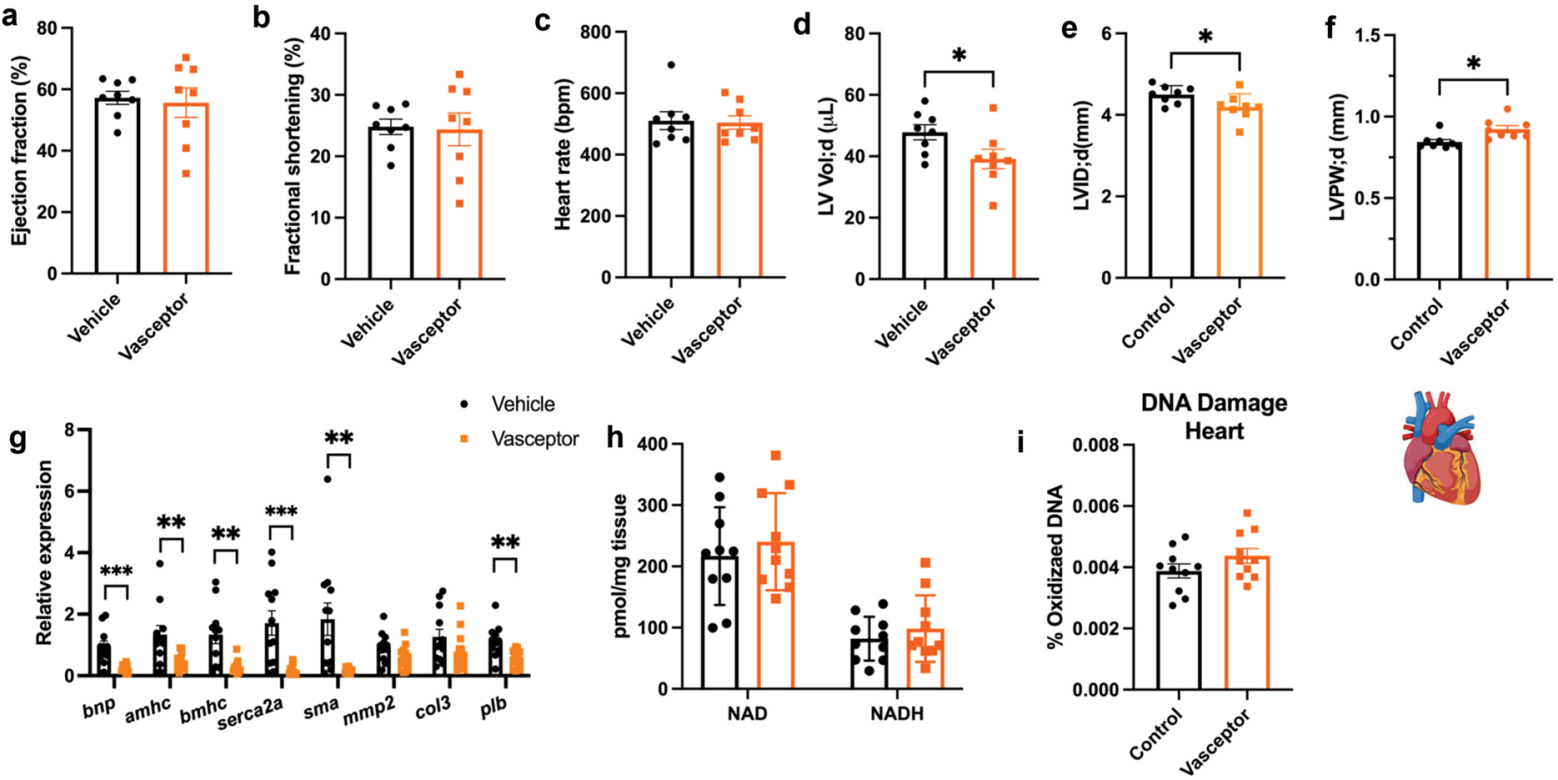
Transdermal curcumin improves diastolic function and reduces expression of fetal genes in myocardium. To determine effects of VASCEPTOR® on cardiovascular health, we performed echocardiogram studies prior to sacrifice (*n* = 8 per group). **a–c** No differences were observed in several cardiac parameters, including ejection fraction (%), fractional shortening (%) or heart rate **d–f** However, during diastole, LV Vol and LVID (LVID;d) were significantly reduced (*p* < 0.05), while LVPW (LVPW;d) was increased (*p* = 0.01) by topical curcumin treatment. **g** When assessing gene expression in myocardium by qPCR, a significant decrease in a number of early fetal genes re-expressed was observed, including *bnp* (*p* = 0.0017), *αmhc* (*p* < 0.05), *βmhc* (*p* = 0.001), *Serca2a* (*p* = 0.0015) and *skeletal muscle actin* (*p* = 0.009), while *phospholamban* (*plb*) was reduced in VASCEPTOR®-treated mice (*p* < 0.05). **h–i** However, there was no effect of treatment on either NAD+ concentration or 8OHdG levels, a marker of DNA damage, in cardiac tissue. Statistics for body weight and ITT were performed via independent samples *t* test. Bar graphs represent the mean ± S.E.. *p < 0.05, **p < 0.01, ***p < 0.001. Figure was generated with assistance from Biorender

To determine effects of VASCEPTOR® on cardiovascular health, we performed echocardiogram studies prior to sacrifice. No differences were observed in several parameters, including ejection fraction (%), fractional shortening (%) or heart rate (Fig. 4a–c). However, during diastole, LV Vol and LVID (LVID;d) were significantly reduced (Fig. 4d–e; *p* < 0.05), while LVPW (LVPW;d) was increased (Fig. 4f; *p* = 0.01) by TD curcumin. This was accompanied by a significant suppression in hypertrophy-related reemergence of early fetal gene expression in myocardium, including *bnp* (*p* = 0.0017), *αmhc* (*p* < 0.05), *βmhc* (*p* = 0.001), *Serca2a* (*p* = 0.0015) and *skeletal muscle actin* (*p* = 0.009), but not in *mmp-2* or *Col3* (Fig. 4g). However, *plb*, which encodes for phospholamban, a key regulator of calcium handling, was reduced in myocardium from VASCEPTOR®-treated mice (Fig. 4g; *p* < 0.05). However, there was no effect of treatment on either NAD+ concentrations or 8OHdG levels in myocardium (Fig. 4h–i).

### Transdermal curcumin modulates expression of pro-aging markers in multiple tissues

**Fig. 5:**
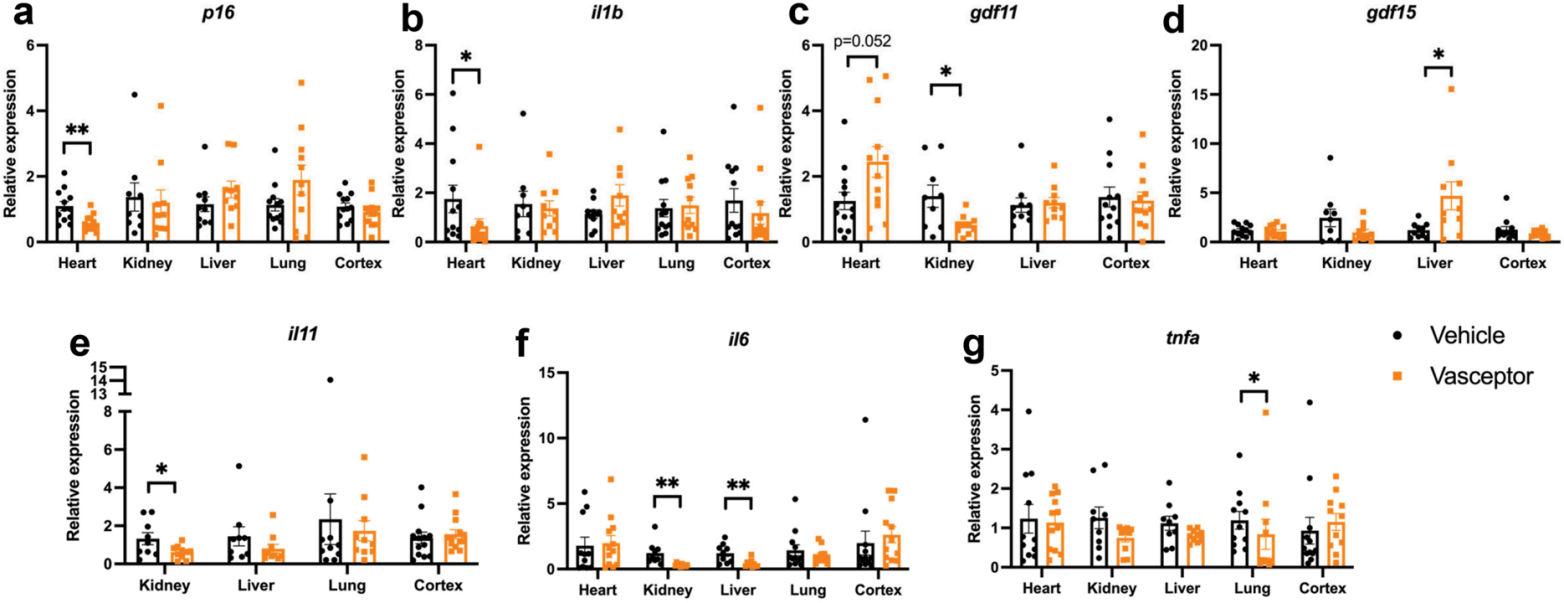
Transdermal curcumin modulates expression of pro-aging markers in multiple tissues. Gene expression of a number of transcripts relevant to age-related processes, such as inflammation, metabolism and senescence in several tissues (heart, kidney, liver, lung, cortex), was examined (*n* = 9–10 per group, per tissue). **a–b** Consistent with the apparent benefits observed in cardiac function, *p16* and *il1b* expression was significantly reduced by VASCEPTOR® in heart, but not elsewhere. *il-1β* was also reduced in heart (*p* < 0.05) with curcumin, but not elsewhere. **c–d** Meanwhile, *gdf11* expression tended to be increased in heart only (*p* = 0.052), but was lower in kidney, while *gdf15* was increased only in liver (*p* < 0.05). **e–g** Lower *il11* expression was detected in kidney of VASCEPTOR®-treated mice (*p* < 0.05), while *il6* was reduced in kidney and liver (*p* < *0*.01). Expression of *tnfα* was significantly reduced in lung (*p* < 0.05), but not elsewhere. Bar graphs represent the mean ± S.E. *p < 0.05, **p < 0.01. Figure was generated with assistance from Biorender

We further assessed a number of transcripts relevant to age-related processes, such as inflammation, metabolism and senescence in several tissues, including heart. Consistent with the apparent benefits observed in cardiac function, p16 expression was significantly reduced by VASCEPTOR® in heart (Fig. 5a; *p* < 0.05). Expression of *il-1β* was also reduced in heart (Fig. 5b; *p* < 0.05) with curcumin but was not significantly different in any other tissues assessed. Meanwhile, *gdf11* expression was increased in heart only (Fig. 5c; *p* = 0.033) while *gdf15* was increased only in liver (Fig. 5d; *p* < 0.05). Lower *il11* expression was detected in cortex and kidney in VASCEPTOR®-treated mice (Fig. 5e; *p* < 0.05), while *il6* was reduced in kidney and liver (Fig. 5f; *p* < 0.05). Expression of *tnfα* was also significantly reduced in lung (Fig. 5g; *p* < 0.05), but not elsewhere.

### Transdermal curcumin restores normal blood pressure following an L-NAME challenge

**Fig. 6:**
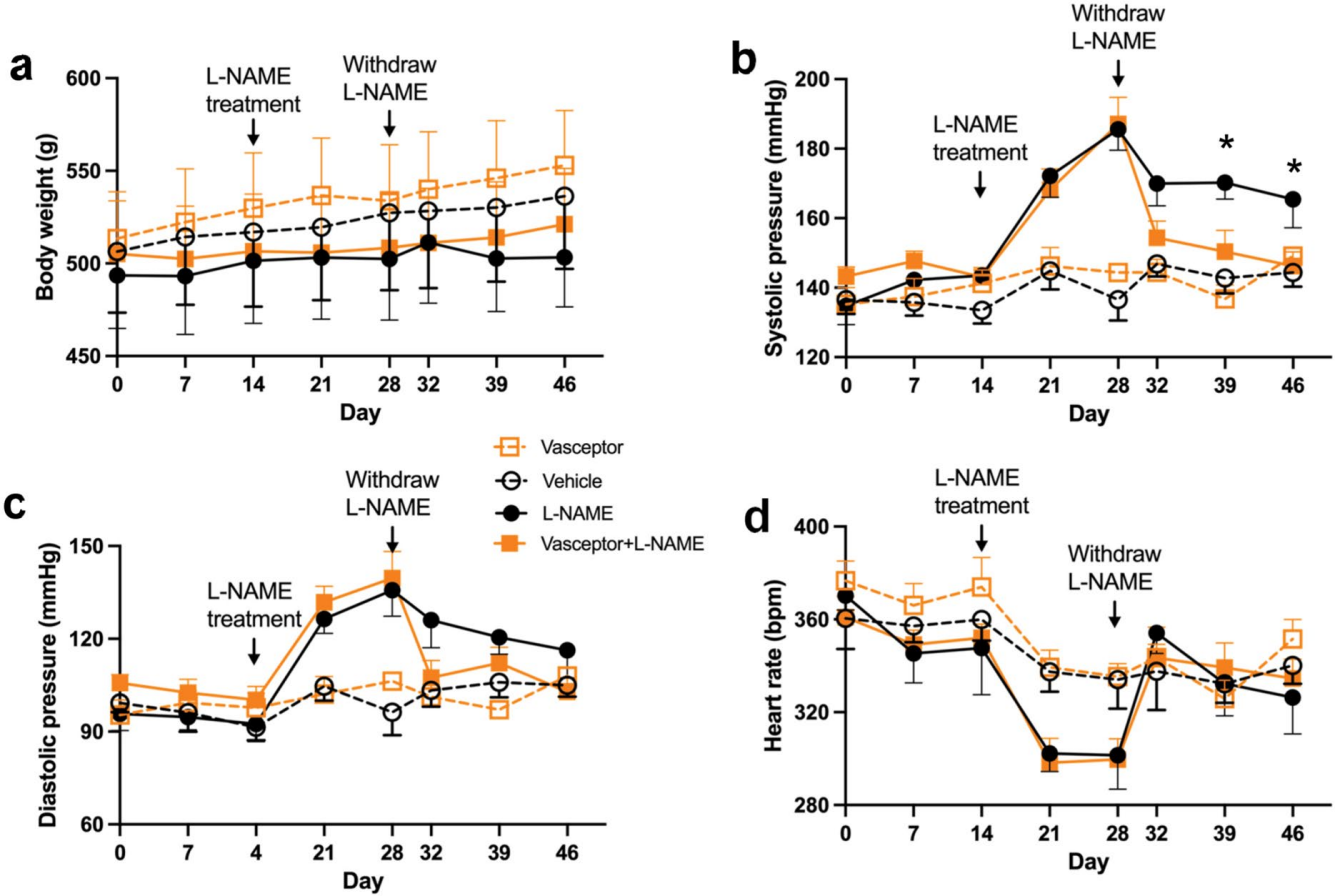
Transdermal curcumin restores normal blood pressure following an L-NAME challenge. We tested the ability of VASCEPTOR® treatment to either confer protection or rescue blood pressure dysregulation in a chemically induced rat model of hypertension. The study was approximately 6 weeks in duration and consisted of a 2-week pre-treatment with VASCEPTOR® or Vehicleprior to L-NAME administration, followed by 2 weeks of L-NAME treatment and then a subsequent 2-week withdrawal (*n* = 7–8 per group). During this time, blood pressure was serially monitored by tail-cuff readings. **a** Body weight was significantly reduced by L-NAME treatment but there were no treat × L-NAME interactions. **b–d** Introduction of L-NAME to the drinking water for 2 weeks resulted in a rapid rise in systolic blood pressure (SBP) and diastolic blood pressure (DBP), along with a reduction in heart rate regardless of treatment. Upon L-NAME withdrawal, heart rate rapidly returned to control levels in both groups, but restoration of normal SBP was only observed in VASCEPTOR®-treated rats by day 7 and 14, respectively. Data were assessed by two-way ANOVA and followed up via planned contrasts when appropriate. Line graphs represent the mean ± S.E. *p < 0.05

Given the purported links of curcumin to the vascular endothelium, we finally set out to test the ability of VASCEPTOR® treatment to preserve blood pressure in an L-NAME induced rat model of hypertension. VASCEPTOR® was given prophylactically 2 weeks prior to L-NAME administration during which time body weight and cardiovascular parameters were similar between treated and control groups (Fig. 6a). Introduction of L-NAME to the drinking water for 2 weeks resulted in diminished weight gain and a rapid rise in systolic blood pressure (SBP; Fig. 6b) and diastolic blood pressure (DBP; Fig. 6c), along with a reduction in heart rate (Fig. 6d), regardless of treatment. Upon L-NAME withdrawal, heart rate rapidly returned to control levels in both groups, but restoration of normal SBP (Fig. 6b) was only observed in VASCEPTOR® treated rats by day 7 and 14, respectively, while DBP tended to recover faster in treated animals versus controls, where signs of hypertension persisted for the duration of the study.

## Discussion

The emergence of evidence that aging hallmarks can be modulated by a plethora of natural compounds, repurposed drugs and more recently by designer molecules and strategies developed to target discrete cellular processes has helped to usher in a new era of gerotherapeutics to delay aging [7, 8]. Natural compounds in particular have been intriguing as supplements for promoting health, as these agents are generally considered inexpensive and assumed safe for use. Unfortunately, a number of the most promising botanical compounds, including turmeric, berberine, quercetin or epigallocatechin-3-gallate (EGCG), lack convincing data in support of efficacy and suffer from notoriously poor oral bioavailability, which may be a major factor in limiting their therapeutic potential [35–37]. While such constraints may be able to be partially overcome by consuming daily, high doses of these compounds as supplements, uptake remains limited and can be accompanied by risks of gastrointestinal symptoms and other limiting side effects.

Curcumin, which is an ancient spice, is particularly intriguing, as it has been touted for its medicinal properties for centuries and remains one of the most commonly consumed dietary supplements for a number of intended ailments [38–44]. However, consistent with prior studies that have attempted to identify strategies to overcome its poor uptake [35–37], we find that oral curcumin administration fails to result in detectable levels of bioactive compound or its metabolites in blood. Nevertheless, an extensive literature exists in both in vitro models and in animal models administered sufficiently high doses of certain formulations that strongly support that curcumin can effectively modulate a number of identified processes in the in vitro or in vivo setting linked to metabolism, inflammation, oxidative stress, nutrient sensing and endothelial function among others [45–47]. However, a single dose-matched topical application of VASCEPTOR® rapidly and effectively penetrated the skin and circulation, resulting in readily detectable levels in plasma, tissues and blood cells, that persisted for at least 6 h in some animals. Indeed, the transdermal route allows for curcumin, and potentially many other promising bioactive compounds to bypass the multitude of barriers encountered to reach circulation when taken orally, including the acidic gastric environment, microbial metabolism, absorption per se and first pass clearance by the liver, which collectively impede the ability of curcumin or bioactive metabolites to enter the blood stream at concentrations likely required for efficacy. Thus, at least based upon these data, transdermal curcumin may have potential to be a safe and effective gerotherapeutic for chronic administration.

Assuming topically administered curcumin can indeed achieve therapeutic levels in humans, as the preclinical data in this study suggest, the potential implications for aging and its hallmarks could be highly impactful. Indeed, pleiotropic actions have been attributed to curcumin, including modulating key processes and pathways that are central to inflammation [48]. Dysregulation in inflammatory pathways and processes are well known to trigger oxidative damage and deficits in other aging hallmarks, thereby contributing to onset of endothelial dysfunction/vascular senescence, neuroinflammation, among many other ailments [43, 49–51]. Inflammation and oxidative stress can set in motion a series of linked reactions including activation of macrophages and CD38 expression, an enzyme that breaks down NAD+ , which is essential for energy production by the mitochondria and sirtuin enzymatic activities [52]. However, curcumin has been shown to prevent and reverse endothelial dysfunction [53–56] and neuroinflammation [50] including opposing aberrant activation of macrophages, limiting ROS production, reversing mitochondrial dysfunction [36, 57] and interacting with some longevity-related signaling mediators and stress-response pathways Moreover, our data here demonstrate that topical curcumin can help to restore cardiovascular function in rodent models. These effects are consistent with reports that curcumin not only acts on the major pathways that trigger inflammation but also can stimulate nitric oxide (NO) production in the endothelium. Indeed, vascular homeostasis is significantly controlled through NO production in the vascular endothelium, which is known to be induced by curcumin [18, 58], a process maintained and regulated through the coupled activity of SIRT 1 and eNOS [59]. Moreover, unlike many biologics, curcumin activity seems to occur without disabling essential biological processes that can ultimately result in triggering autoimmune responses and other unwanted consequences.

As a compelling proof-of-concept, we show here that just a 12-week intervention with VASCEPTOR® in 20-month-old mice (~ 60–65-year-old human equivalent) led to a profound improvement in a number of health span indices, particularly as it pertains to physical function-related parameters, including coordination, stamina, cardiac function and frailty status. Indeed, loss of coordination, stamina, fatigue and decreasing capacity for exercise is a consistent manifestation of normal aging. This effect of late-onset curcumin is somewhat reminiscent of rapamycin [6], which also has immune-modulating effects, and remains the most robust gerotherapeutic, with the most compelling evidence of efficacy among compounds initiated at middle to older ages. Moreover, VASCEPTOR® modulated a number of transcripts across diverse tissues related to inflammation and senescence as well as myocardial-related genes, further supporting its systemic benefits. Of note was a significant reduction in kidney *il11* expression, a cytokine recently linked to lifespan in mice [60]. These effects on inflammation and senescence coincide with improved health span, and thus it stands to reason may have contributed to their improvements, though the precise pathway(s) and processes most important to these effects will require further investigation in the in vivo context.

Likewise, VASCEPTOR® helped to rapidly normalize blood pressure following an L-NAME challenge in a rat model of hypertension, further implicating the therapeutic potential of transdermal curcumin in both normal aging and pathologic states. These data are consistent with human trials which noted improved endothelial function following 12-week curcumin supplementation, which was attributed to increased NO production. However, this oral dose did not affect oxidative stress or inflammatory markers, perhaps owing to an inability to achieve the therapeutic levels needed via the oral route to target these pathways [18].

Beyond the promise of better harnessing the therapeutic potential of curcumin via the transdermal route, alone or combined with other known or suspected gerotherapeutics, such as metformin, SGLT2 inhibitors or GLP-1 medications, this study also raises the specter of developing even more potent transdermal gerotherapeutics via novel combinatorial formulations with other promising natural compounds whose efficacy has also been stymied by ineffective oral delivery. Such combinations could be optimized to modulate complementary cellular and molecular processes in an additive or synergistic fashion via a continuous or precision-type regimen to more effectively target multiple aging hallmarks simultaneously with the goal of delaying aging, its diseases and extending health span. Another distinct advantage of curcumin and other related natural compounds from a translational perspective is that they can be rapidly deployed since they are not subject to the same FDA scrutiny as novel pharmaceuticals, whose developmental timeline can take years to progress through clinical trials. Thus, transdermal strategies devised to deliver natural compounds capable of improving and/or preserving health span represent a promising platform to rapidly establish an effective, tangible gerotherapeutic for an older population in urgent need of such options to combat the manifestations of aging.

In summary, this proof-of-concept study demonstrates that transdermal delivery of VASCEPTOR®, a concentrated curcumin formulation designed to be skin penetrant, confers superior bioavailability in circulation to oral administration. Moreover, we show that VASCEPTOR® can mitigate dysfunction in a rat model of hypertension, while a short-term, late-life intervention with VASCEPTOR® led to markedly improved health span metrics across multiple domains. Importantly, no adverse effects were evident via twice weekly administration in either a mouse or rat model. However, there are important limitations here that can only be addressed by additional studies. First, we elected to focus only on male mice in this endeavor since we suspected that inflammation would be an important target of treatment, and most, but not all agents tested in ITP with anti-inflammatory effects, such as NDGA, aspirin and 17α-estradiol, seem to preferentially benefit males [2, 61]. Thus, the ability of TD curcumin to improve health span and other biomarkers in females remains to be determined. Future studies are also needed to determine if benefits of VASCEPTOR® on aging can be enhanced by further refining the dosing regimen and/or intervening at an earlier age for a longer duration. Along these lines, Pk properties of VASCEPTOR® are limited here to younger animals and will need to be more extensively assessed in both blood and solid organs following acute and chronic TD dosing in middle and older ages of both sexes to help guide optimal dosing strategies that are safe. To our knowledge, this study represents the first example of a transdermally delivered gerotherapeutic and suggests that this strategy represents a highly tractable and viable approach to harness for development of rapidly deployable and efficacious treatments to target human aging.

## Data Availability

Data are freely available from the corresponding author upon reasonable request.
